# Effects of harvest, fire, and pest/pathogen disturbances on the West Cascades ecoregion carbon balance

**DOI:** 10.1186/s13021-015-0022-9

**Published:** 2015-05-20

**Authors:** David P Turner, William D Ritts, Robert E Kennedy, Andrew N Gray, Zhiqiang Yang

**Affiliations:** 1 Department of Forest Ecosystems and Society, Oregon State University, 97331 Corvallis, OR USA; 2 College of Earth, Ocean, and Atmospheric Sciences, Oregon State University, 97331 Corvallis, OR USA; 3 USDA Forest Service, Pacific Northwest Station, 97331 Corvallis, OR USA

**Keywords:** Forests, Carbon, Net ecosystem production, Net ecosystem exchange, Net ecosystem carbon balance, Disturbance, West Cascades ecoregion

## Abstract

**Background:**

Disturbance is a key influence on forest carbon dynamics, but the complexity of spatial and temporal patterns in forest disturbance makes it difficult to quantify their impacts on carbon flux over broad spatial domains. Here we used a time series of Landsat remote sensing images and a climate-driven carbon cycle process model to evaluate carbon fluxes at the ecoregion scale in western Oregon.

**Results:**

Thirteen percent of total forest area in the West Cascades ecoregion was disturbed during the reference interval (1991-2010). The disturbance regime was dominated by harvesting (59 % of all area disturbed), with lower levels of fire (23 %), and pest/pathogen mortality (18 %). Ecoregion total Net Ecosystem Production was positive (a carbon sink) in all years, with greater carbon uptake in relatively cool years. Localized carbon source areas were associated with recent harvests and fire. Net Ecosystem Exchange (including direct fire emissions) showed greater interannual variation and became negative (a source) in the highest fire years. Net Ecosystem Carbon Balance (i.e. change in carbon stocks) was more positive on public that private forestland, because of a lower disturbance rate, and more positive in the decade of the 1990s than in the warmer and drier 2000s because of lower net ecosystem production and higher direct fire emissions in the 2000s.

**Conclusion:**

Despite recurrent disturbances, the West Cascades ecoregion has maintained a positive carbon balance in recent decades. The high degree of spatial and temporal resolution in these simulations permits improved attribution of regional carbon sources and sinks.

## Background

Net uptake of carbon by forests provides a significant offset to anthropogenic carbon emissions at the global [1], national [2, 3], regional [4], and landscape [5] scales. However, forest carbon sinks are vulnerable to disturbances in the form of harvesting, fire, and pest/pathogen outbreaks. At the regional scale, we have a poor understanding of the relative contribution of these disturbances to overall carbon budgets [6], but such knowledge is important in understanding how the carbon cycle is responding to on-going management and climate change [7, 8]. It is also critical for developing policies for greenhouse gas mitigation through altered land use [9].

Forest disturbances are a strong determinant of carbon stocks and fluxes on both managed and unmanaged landscapes [5, 10]. Clear-cut harvesting, as is commonly practiced in coniferous forests of western Oregon, shuts down the photosynthetic carbon sink and increases the carbon source from heterotrophic respiration of harvest residues. Partial harvests for thinning likewise induce a near term reduction in carbon sequestration [11]. Wildfire is similar to harvesting in reducing carbon uptake, but has a longer term impact on heterotrophic respiration because of the slow conversion of snags to more readily decomposed woody debris on the ground [12, 13]. Pest/pathogen outbreaks reduce leaf area and leave slow decomposing snags, thus altering ecosystem carbon flux for decades [14, 15]. Spatially-explicit carbon cycle assessments in the western U.S. have generally emphasized effects of harvests and fire [16–18] and not explicitly captured the impact of slow pest/pathogen disturbances.

Satellite remote sensing, particularly from the Landsat series of sensors (~30 m resolution), offers the opportunity to monitor forest disturbances [19, 20]. In combination with spatially-distributed ecosystem process models that simulate carbon cycle responses to specific disturbances, remote sensing data can be used to map and monitor forest carbon stocks and flux [21, 22]. Here, we take advantage of a new Landsat-based time series analysis of forest disturbance (LandTrendr) [23, 24] and a well-established modeling infrastructure for simulating regional carbon flux based on the Biome-BGC carbon cycle process model [25] to quantify carbon cycle impacts of harvesting, fire, and pest/pathogen outbreaks on forests of the West Cascades (WC) ecoregion in the Northwestern U.S. We make extensive use of plot scale and aggregated U.S.D.A. Forest Service Forest Inventory and Analysis (FIA) data [26, 27] for calibration and validation of both LandTrendr and Biome-BGC.

We quantify several distinct carbon fluxes [28] at the ecoregion scale. Net ecosystem production (NEP) is the balance of net primary production (NPP) and heterotrophic respiration (R_h_). It reflects ecosystem metabolism as it responds to variation in weather, and to disturbance events. Net ecosystem exchange (NEE) is the absolute vertical flux of CO_2_ over a given geographical domain. This is the flux as “seen” by a continental to global scale inversion, *e.g*. [29]. In addition to NEP and direct fire emissions, it includes river/stream evasion as well as emissions associated with harvested products [30]. Net ecosystem carbon balance (NECB) refers to the absolute change in carbon stocks, and is affected by NEP as well as removals in the form of harvested products, lateral transfers of dissolved organics, and by direct fire emissions. NECB is the equivalent of carbon sequestration as would be relevant to offsetting fossil fuel emissions. For the purposes of comparisons here, we report NEE using the same convention as with NEP and NECB, i.e. a positive value is a carbon sink. The capacity to isolate these fluxes is required to fully understand the role of forests and forest management in regulating the atmospheric CO_2_ concentration.

## Results

### Domain characterization

**Fig. 1:**
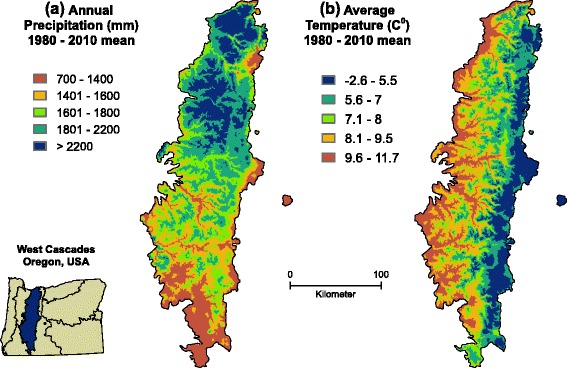
Climate in the study area: **a** annual precipitation, **b** annual average temperature

**Fig. 2:**
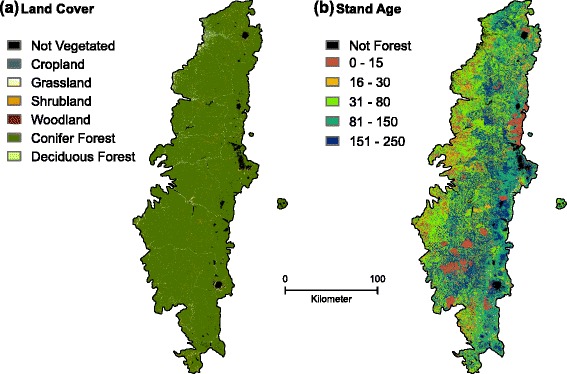
The West Cascades domain: **a** land cover, **b** stand age

**Fig. 3:**
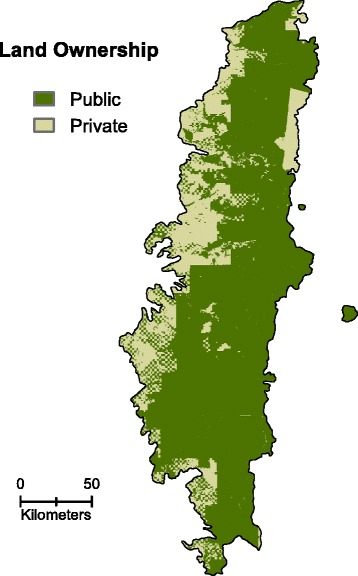
The distribution of public and private forestland

Our study domain was the West Cascades ecoregion in western Oregon, U.S.A. It is characterized by a strong elevation gradient from west to east, with corresponding gradients in temperature and precipitation (Fig. 1). Land cover is predominantly conifer forest (Fig. 2a). Forest stand age tends to be <60 on the low elevation private lands that are managed for wood production (Figs. 2b, and 3). On public lands at higher elevations, there is a broader range of stand ages.

### Disturbance patterns

**Fig. 4:**
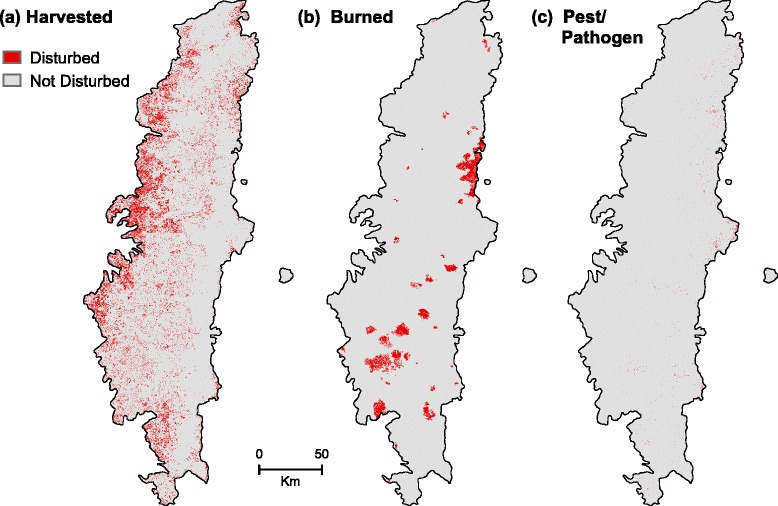
Location of disturbances: **a** harvest, **b** fire, **c** pest/pathogen

**Fig. 5:**
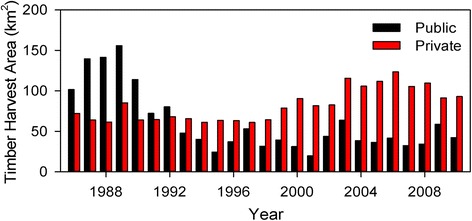
Area harvested by year for public and private forestland (1986-2010)

**Fig. 6:**
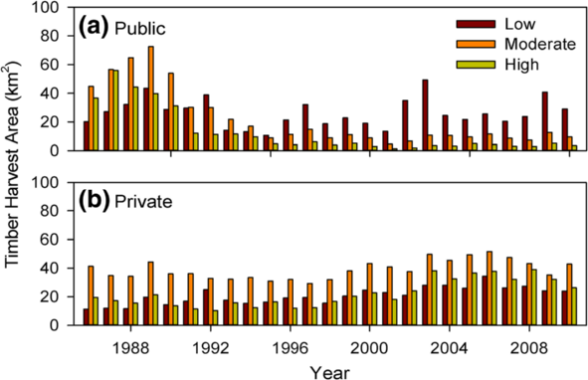
Trends in harvest magnitude (1986-2010): **a** public forestland, **b** private forestland

The total proportion of the study area that was disturbed during the 1991-2010 interval was 13 %. The proportion of total disturbed area attributed to harvest was 59 %. The corresponding proportion for fire was 23 %, and for pest/pathogen outbreak was 18 %. The location of the harvests have been predominantly on lower elevation private forestland (Fig. 4a). The time series of annual area harvested shows a decrease on public lands in the late 1980s and an increase on private lands in the most recent decade (Fig. 5). On public lands, there was a shift from stand replacing harvests to partial harvests whereas on private forestland stand replacing harvests were most common over the whole time series (Fig. 6).

**Fig. 7:**
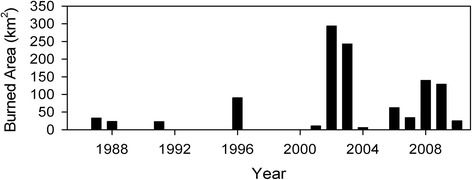
Area burned by year for total forestland (1986-2010)

The incidence of fire was low in the 1980s and 1990s but increased appreciably in the 2000s (Fig. 7), with fires located at both high and low elevations (Fig. 4b). The overall proportion of fires at high, medium, and low intensity was 28 %, 33 %, and 40 % respectively. These proportions did not change much from year to year.

Areas of pest/pathogen disturbance occurred primarily at high elevations (Fig. 6c). Intensity tended to be higher in the years with relatively large areas disturbed.

### Carbon flux

**Fig. 8:**
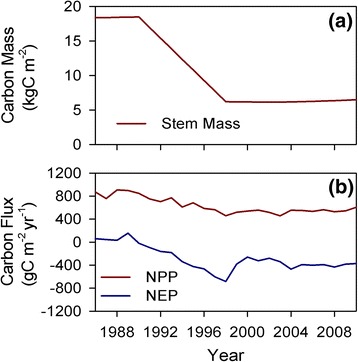
Representative simulation results for a disturbed stand. Conditions are high intensity and short duration pest/pathogen disturbance beginning in 1990 at a mid-elevation site. **a** Stem mass, **b** Net Primary Production (NPP) and Net Ecosystem Production (NEP)

In our stand-level simulations of pest/pathogen disturbance, NPP falls in parallel with the drop in stem and foliar biomass and begins to recover after the year of maximum intensity (*e.g*. Fig. 8). NEP correspondingly decreases, falling below zero in the case of a short, high intensity slow disturbance.

**Fig. 9:**
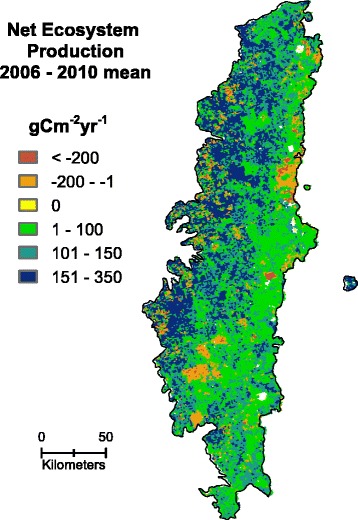
Net ecosystem production over the West Cascades ecoregion (mean for 2006-2010)

**Fig. 10:**
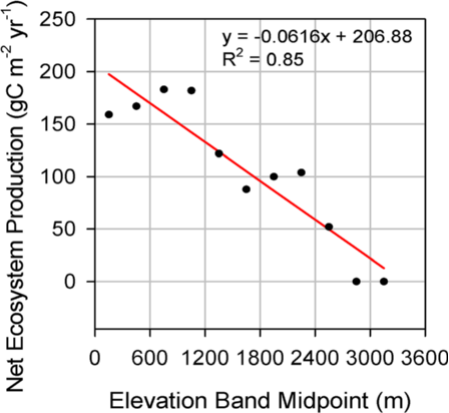
Relationship of mean Net Ecosystem Production (2006-2010) to elevation band

In the ecoregion-wide simulations, the majority of the surface area of the WC ecoregion had a positive NEP during the study period (*e.g*. Fig. 9). NEP tends to decrease as elevation increases (Fig. 10) because of decreasing rates of wood productivity associated with a shorter growing season, and a shift towards older, slower growing, age classes on public lands (Fig. 2b). Carbon sources (negative NEP) are indicated in areas recently burned or harvested.

**Fig. 11:**
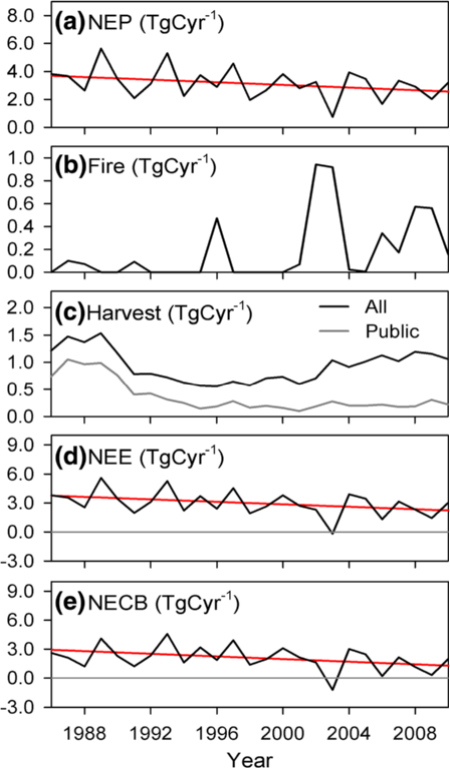
Annual ecoregion totals for (**a**) net ecosystem production (NEP), (**b**) fire emissions, (**c**) harvest removals, (**d**) net ecosystem exchange (NEE), (**e**) net ecosystem carbon balance (NECB)

The ecoregion total NEP was also generally positive (Fig. 11). Total direct fire emissions increased in the 2000s relative to the 1990s, but only exceeded NEP in 2003. Over the 1991-2010 interval, harvest removals offset 28 % of NEP and direct fire emissions offset 7 % of NEP.

The interannual variation in NEE is a function of both NEP and direct fire emissions. In 2003, which was a high fire year (Fig. 7), high temperatures and soil drought reduced NPP more than R_h_, and hence reduced NEP. Direct fire emissions were also relatively high, causing a dip in NEE (Fig. 11).

The ecoregion total NECB averaged 1.9 (SD = 1.3) TgC yr^-1^ over the 1991-2010 interval. Only in 2003 did it fall below zero. Thus, the ecoregion has been a sustained sink for atmospheric CO_2_ in recent decades. The area weighted mean NECB (1991-2010) for public lands was 65 gC m^-2^ year^-1^ compared to 8 gC m^-2^ year^-1^ on private forestland, reflecting a proportionally lower harvest rate on public lands in recent years.

## Discussion

### Harvest

Since much of the WC ecoregion is public forestland, the reduction in harvests associated with implementation of the Northwest Forest Plan (NWFP) in the early 1990s [31] had a notable impact on the overall rate of harvesting [24, 32]. The effect has been stabilization of a long-term decline in the proportion of public lands that is in the old-growth condition [33].

The rate of harvest on private forestland also shifted over the course of the study period, but in this case it was an increase that reflected a period of economic growth and high demand for wood products. The observed harvest rate of 1.5 % per year on private forestland over the 2000-2010 period is consistent with the 45–60 year rotation that is common in the area.

The notable shift on public lands from high intensity (clear-cut) harvesting to low and moderate intensities (thinning) reflects policy changes. Beginning in the 1990s, litigation largely prevented further harvesting of old growth stands. However, quotas established by the NWFP did allow for a low level of harvesting. Thinning of young to mature stands thus became the standard practice [34, 35], as reflected in the LandTrendr observations. On private lands that are managed primarily for wood production, the practice of clear-cut harvesting has remained the standard.

### Fire

The fire regime in the WC ecoregion has varied over the last several hundred years. Fire scar data and tree age class distributions indicate a period of high fire about 1500, possibly associated with a relatively warm climate [36, 37]. During the settlement era in the late 19th century, the incidence of fire increased because of anthropogenic factors. That period was followed by a large decrease in the incidence of fire in the 20th century, associated with successful fire suppression. Most recently, an increase in the incidence of forest wildfire has been noted over much of the western U.S. and attributed in part to climate warming [38]. The post-2000 increase in area burned in this study is consistent with this broad pattern.

**Fig. 12:**
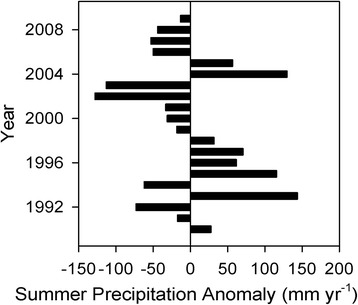
Anomaly in May 1 through September 30 precipitation

The sharp increase in the area burned in the WC ecoregion in the 2000s (3.6 % per year) compared to the 1990s (0.4 % per year) is associated with a 20 % decrease in mean May through September precipitation (Fig. 12). There was also a minor increase (0.2 °C) in mean May through September temperature. This natural experiment in interdecadal climate variation mimics to some degree the summer precipitation trends expected in the region for the 21st Century [39]. The observations here lend support to projections of an increased incidence of fire over the course of the 21st Century in the Cascade Mountains [40, 41]. Note that detecting relationships between temporal trends in burned area and climate is potentially confounded by changes in policy with respect to managing fire [42].

### Pests/pathogens

Our trajectory-based LandTrendr algorithm is complimentary to the U.S. Forest Service airborne surveys for pests/pathogens in that it better resolves spatial heterogeneity and is sensitive to severity [43]. The duration of the slow disturbances is also informative with respect to identifying the relevant organisms: western spruce budworm show a more consistent long-term decline in the reference spectral vegetation index compared to mountain pine beetle [44].

The elevated incidence of pest/pathogen disturbances around 1990 is associated with a multiyear outbreak of western spruce budworm at relatively high elevations. This outbreak was widespread over the western U.S. [45, 44]. After 1995, the annual area disturbed became stable, presumably at an endemic population level characteristic of the native pests/pathogens. Meigs et al. [44] examined the incidence of area in the Cascade Mountains subject to pest/pathogen impacts and later burned. While that sequence was not uncommon, there did not appear to be a strong relationship. Indeed, mountain pine beetle outbreaks in lodgepole pine forests, may reduce the probability of active crown fire [46].

### Carbon flux

NEP. In forest ecosystems, NEP is generally negative after a stand replacing harvest (because NPP is low and R_h_ from decay of residues is high), becomes moderately positive for multiple decades while wood mass accumulates, and then falls to near zero in late succession [47, 48]. Stand-level simulations with Biome-BGC are in agreement with that pattern [17, 49].

The stand age for crossover from carbon source to carbon sink varies widely in the WC ecoregion because of differences in the amount of residues present at the time of stand initiation [50]. The post disturbance pulse of R_h_ and negative NEP is of greater magnitude in the case of harvest but of greater duration in the case of fire because the proportion of the biomass actually burned in a wildfire is often small [51] and standing dead trees (snags) decay relatively slowly [13].

Moderate to low intensity abrupt disturbances (*e.g*. thinning) may also introduce periods of negative NEP [52]. Lost leaf area means a smaller fraction of photosynthetically active radiation is captured by the canopy, and heterotrophic respiration is boosted by the metabolism of the pests/pathogens themselves [53] or by heterotrophic respiration of the increased leaf and fine root litter [54]. In our Biome-BGC simulations, the time to recovery of positive NEP is closely tied to the magnitude of the disturbance.

Here we also treat slow disturbances presumed to be caused by pests/pathogens. These disturbances are similar to fires in leaving large amounts of dead wood biomass behind that are slowly respired by heterotrophs and hence reduce NEP [14].

The most controversial aspect of the generalized trajectory for NEP over the course of succession in the WC ecoregion is the degree to which relatively old stands remain a carbon sink [55, 56]. Given that maritime Douglas-fir trees can survive for over 1000 years [57], and possibly benefit from increasing temperature and CO_2_ concentration, a long term carbon sink might be expected. Mean simulated NEP (2001-2009) for old growth stands in the study area was 70 gC m^-2^ year^-1^, which compares to and eddy covariance based mean of 49 gC m^-2^ year^-1^ (1998-2008) at the Wind River old growth Douglas-fir site [58].

Interannual variation in climate variables drives the large interannual variation in ecoregion-wide NEP in our simulations. Observations at eddy covariance flux towers in the Cascade Mountains indicate that both gross primary production and ecosystem respiration contribute to interannual variation in NEP [58, 59], with smaller carbon sinks associated with warmer, drier years. Tree ring observations find that relatively warm years increase tree growth at high elevations [60, 61] and reduce it at mid to low elevations [62] in the Cascade Mountains. Thus, spatially distributed simulations are needed to evaluate if projected climate change driven warming and possible summer drying in the Pacific Northwest [39] would act to increase or decrease ecoregion mean NEP [7, 63].

NEE. The declining trend of NEE between the 1990s and 2000s was driven by both a decrease in NEP (dominant factor) and an increase in direct fire emissions. Schwalm et al. [7] also report a drop in regional NEP associated with turn of the century drought in western North America. Previous Biome-BGC simulations in Oregon suggest that both NPP and R_h_ have declined in recent decades, with a greater decline in NPP [64]. However, there remains considerable uncertainty about the impact of changing climate and CO_2_ concentration on regional tree growth. Long term observations at 21 temperate and boreal zone eddy covariance flux towers support a broadly realized CO_2_ benefit on forest water use efficiency [65], which may offset the impacts of lower summer precipitation.

By estimating NEE, we are in a position to compare our ecoregion fluxes with independent scaling approaches. Our mean NEE for 2004 was 148 gC m^-2^ years^-1^ (a carbon sink), much larger that an estimate from the CarbonTracker inversion [29, 66] of 18 gC m^-2^ years^-1^. Differences of this magnitude have been observed in other NEE comparisons [67, 68] and point to the need to reconcile results from alternative scaling approaches [69]. LandTrendr could potentially deliver detailed information on the disturbance regime over much larger domains than the present study, thus opening the possibility for improved regional carbon budgets.

NECB. The positive NECB for the ecoregion using our flux scaling approach is consistent with carbon sinks in western Oregon reported using other modeling approaches [70] as well as inventory based approaches [35, 71, 72]. An additional forest sector sink is associated with harvested wood removals that accumulate in long-lived wood products [73] but is not estimated here. The high carbon accumulation rate on public lands, as driven by a policy-based lower harvest rate, supports the inclusion of carbon sequestration in the suite of ecosystem services to be considered in public forestland management.

Forest carbon stocks and flux are now widely recognized as highly relevant to mitigating the on-going rise in atmospheric CO_2_ [74] and the combined remote sensing/modeling approach described here offers the opportunity to monitor forest carbon budgets at the fine spatial and temporal resolution that may be needed for quantifying carbon sinks. Ongoing efforts to characterize uncertainty associated with the remote sensing of disturbance and with stand-age-specific carbon fluxes (*e.g*. [75]) will increase the policy relevance of these carbon flux maps. The end points of our analysis are also the prerequisites for a realistic landscape simulation into the future that accounts for climate change and land use [76].

## Conclusions

Harvests are the dominant form of disturbance in the West Cascades ecoregion, followed by fire, and pests/pathogens. The majority of the WC landscape has a positive NEP most of the time, and annual total NEP has been positive in recent decades. In high fire years, ecoregion total NEE can fall below zero (become a source) because of low NEP and high direct fire emissions. Harvest removals offset 4 times more NEP than do direct fire emissions. The sustained carbon accumulation on the West Cascades land base contributes to the regional forest carbon sink.

## Methods

### Overview

Our NEP/NECB scaling methods and validation results for applications in Oregon, Washington, and California have been reported in Turner et al. [18, 21, 22, 64] and Law et al. [77, 78]. These previous studies considered only stand-replacing disturbances and employed simple Landsat-based change detection algorithms to specify the approximate year of disturbance. Here we added treatment of pest/pathogen outbreaks (slow disturbance), allowed for multiple disturbance intensity classes, and accounted for sequential disturbance events based on an annual time series of Landsat data. To quantify NEE, we added a previously derived estimate of emissions from harvested products (wood and crops) to NEP and fire emissions.

### NEP modeling

Our primary tool for scaling carbon flux across the ecoregion is the Biome-BGC carbon cycle process model [25]. The model has a daily time step and is run over multiple years to simulate succession. Simulated carbon cycle processes include photosynthesis, autotrophic respiration, heterotrophic respiration, plant C allocation, and mortality. Simulated C pools include stemwood, coarse roots, fine roots, foliage, litter, coarse woody debris, snags, and soil organic matter. A water balance is calculated based on the Penman-Montieth formulation of evapotranspiration. For each model run, there is a spin-up using a 25-year repeating loop of climate data to bring the soil carbon pool into near equilibrium with the climate. At its end, one or two disturbance events are prescribed by year, type, duration, and intensity to bring the simulation up to the current condition.

A look-up table determines the partitioning of the extant carbon stocks existing at the time of disturbance into removals, direct fire emissions, and transfers of necromass from one ecosystem component to another, thus maintaining ecosystem mass balance (Table 1). The disturbance history of a stand is prescribed in terms of one or two disturbances based on the record of Landsat imagery.

**Table 1:** Partitioning of biomass pools at the time of disturbance

		Biomass component					
		Leaf/fine root	Stem	Coarse root	Litter	CWD	Snag
Disturbance	Disturbance						
Type	Magnitude						
Harvest	Low	0.25^a^	0.25^b^	0.25^c^			
	Medium	0.55^a^	0.55^b^	0.55^c^			
	High	1.00^a^	1.00^b^	1.00^c^			
Fire^d^	Low	0.125	0.02^e^	0.02^f^	0.66	0.17	0.11
	Medium	0.50	0.03^e^	0.03^f^	0.66	0.22	0.14
	High	1.00	0.05^e^	0.05^f^	1.00	0.39	0.18
Pest/Pathogen	Low	0.30^a^	0.30^g^	0.30^c^			
	High	0.75^a^	0.75^g^	0.75^c^			

Values are proportions of total biomass. CWD = coarse woody debris

^a^Transferred to litter

^b^Stem transferred off-site, associated branches to go coarse woody debris (CWD)

^c^Transferred to coarse woody debris

^d^All transfers are to the atmosphere

^e^Residual mortality is transferred to snag

^f^Residual mortality is transferred to coarse woody debris

^g^Transferred to snag

The version of Biome-BGC used here was adapted from version 4.1.2 [25] to simulate stand-replacing disturbances [77], dynamic allocation over the course of succession [78], and mixed severity fire [17]. To accommodate partial disturbances, the proportional disturbance intensity from satellite data (see below) is applied directly to the live carbon pools (Table 1).

The initiation year, duration, and maximum magnitude are prescribed by LandTrendr in the case of pest/pathogen disturbances. A linear ramp from initiation year to the maximum magnitude year is used to prescribe an annual mortality amount such that the prescribed maximum mortality is achieved in the maximum mortality year (Fig. 8). The stand recovers from disturbance prognostically. Transfers of dead foliar and fine root C are made to the litter pools and transfers of tree C to a standing dead (snag) pool. There is subsequent transfer to coarse woody debris on the ground [79–81]. Partitioning factors at the time of disturbance are based on observations within the region [51, 82–84].

Biome-BGC has 20 cover-type specific ecophysiological parameters that must be specified. For the most part, we adopted the recommendations in White et al. [85] and a representative set of parameters is given in Turner et al. [64]. In the case of the evergreen needle leaf cover type, we did an ecoregion specific adjustment on two of the parameters (the fraction of leaf nitrogen as rubisco, and the mortality fraction) that had been identified in sensitivity analyses as strongly impacting wood mass and NEP. Reference data for these parameter selections were site- and age-specific estimates of net stem growth and wood mass from USDA Forest Service FIA plots [35]. We ran the model at all FIA plot locations (approximated within 500 m to preclude potential disclosure of confidential locations) within the ecoregion to the stand age in the plot data, and compared predicted and observed net stem production using a range of possible parameter values. The optimal value for the mortality parameter was 0.0125 (proportion of biomass per year) and for the fraction of leaf nitrogen as rubisco parameter was 0.035 (unitless).

### Model inputs

Our base land cover dataset (Fig. 2a) is the 2011 National Land Cover Database (NLCD) [86] which is derived from Landsat data. Areas that had been harvested and were classified by NLCD as shrubland were reclassified as forest. The distribution of public and private land (Fig. 3) was from U.S. Geological Survey [87].

The attributes of the disturbance regime were from the LandTrendr analysis of Landsat Thematic Mapper time series data [23, 24]. It captures both abrupt events, such as fire and harvest, and slow (multiyear) disturbance processes caused by pests/pathogens. Each disturbance event is characterized by year, type, magnitude, and duration. The year is indicated by an inflection point in the trajectory of a spectral vegetation index in each pixel-level Landsat time series. The type of disturbance can be harvest (thinning), fire, or pest/pathogen driven. The distinction for abrupt disturbances between fire and harvest is based on reference to the Monitoring Trends in Burn Severity (MTBS) dataset [88]. Variables in the LandTrendr output are reported in a continuous format but here we binned the outputs into 3 magnitude classes for fire and harvest (see Table 1), and 2 magnitude classes (see Table 1), each with 3 possible durations (9, 17, 25 years), for slow disturbances. Bin midpoints are assigned to represent stand age, disturbance magnitude, and disturbance duration.

The LandTrendr analysis covered the interval from 1985 to 2012, but here we used multiyear buffers at the beginning and end when reporting carbon fluxes to limit artifacts associated with identifying inflection points in slow disturbances.

Disturbances previous to 1986 were prescribed on the basis of mapped stand age class from gradient nearest neighbor analysis (GNN) [89]. GNN integrates data from Landsat and Forest Inventory and Analysis plots [26] to estimate stand age. Conifer stands not having LandTrendr-based disturbance since 1985 were binned into 4 age classes and assigned the age class midpoint (45, 80, 150, 250 years). The binning of disturbance magnitude and duration as well as stand age class was necessary to constrain the number of unique disturbance histories within each 1 km climate grid cell. Model runs were made for the 10 most frequent combinations of cover type and disturbance history in each 1 km grid cell, and fluxes were reported as the weighted mean per 1 km^2^ cell. Using this approach, we covered >90 % of the forest area under consideration.

The meteorological inputs to Biome-BGC are daily minimum and maximum temperature, precipitation, humidity, and solar radiation. We obtained 25 years (1986-2010) of climate data at 1 km resolution over our study ecoregion through the North American Carbon Program [90]. The distributed climate data (Fig. 1) are based on interpolations of meteorological station observations using a digital elevation model and general meteorological principles [91–93]. Uncertainty in the interpolations has previously been evaluated in our region and elsewhere [94, 95].

Soil Data Input. Soil texture and depth are specified from US Geological Survey soil maps [96].

### Carbon flux reporting

NEP was calculated as NPP minus R_h_. NEE was NEP minus direct fire emissions and an estimate for emissions from harvested products [97, 68]. This later estimate was made by reference to the change in the stock of previously harvested wood and crop products (based on inputs associated with harvests and product-specific turnover rates) and population distribution [97]. River stream evasion is also a significant term in regional NEE, however much of it derives from CO_2_ originating in R_h_ of soil organic matter that is flushed into streams with the flow of the soil solution [98]. Since our Biome-BGC simulation includes R_h_ of soil organic matter, we did not attempt to additionally account for river/stream evasion here. NECB was calculated as NEP minus fire emissions and harvest removals (from our own simulations). Our NECB did not explicitly account for land use change, but the rate of land use conversion is quite low in the WC ecoregion [72].

## Abbreviations

FIA: Forest inventory and analysis
GNN: Gradient nearest neighbor analysis
MTBS: Monitoring trends in burn severity
NEP: Net ecosystem production
NECB: Net ecosystem carbon balance
NEE: Net ecosystem exchange
R_h_: Heterotrophic respiration
NPP: Net primary production
NWFP: Northwest forest plan
U.S.: United States
USDA: United States Department of Agriculture
WC: West Cascades
